# Effect of family-centered interventions for perinatal depression: an overview of systematic reviews

**DOI:** 10.3389/fpsyt.2023.1094360

**Published:** 2023-06-01

**Authors:** Liping He, Kim Lam Soh, Jiaxiang Yu, Aixiang Chen, Xiujuan Dong

**Affiliations:** ^1^Department of Nursing, Faculty of Medicine and Health Sciences, Universiti Putra Malaysia, Serdang, Selangor, Malaysia; ^2^Department of Nursing, Chang Zhi Medical College, Changzhi, Shanxi, China

**Keywords:** perinatal depression, family-centered interventions, overview of systematic reviews, efficacy, quality

## Abstract

**Objective:**

This study aimed to evaluate and conclude the quality of critically systematic reviews (SRs) of the efficacy of family-centered interventions on perinatal depression.

**Methods:**

SRs of the efficacy of family-centered interventions on perinatal depression were systematically searched in nine databases. The retrieval period was from the inception of the database to December 31, 2022. In addition, two reviewers conducted an independent evaluation of the quality of reporting, bias risk, methodologies, and evidence using ROBIS (an instrument for evaluating the bias risk of SRs), Preferred Reporting Items for Systematic Reviews and Meta-Analyses (PRISMA), AMSTAR 2 (an assessment tool for SRs), and grading of recommendations, assessment, development and evaluations (GRADE).

**Results:**

A total of eight papers satisfied the inclusion criteria. In particular, AMSTAR 2 rated five SRs as extremely low quality and three SRs as low quality. ROBIS graded four out of eight SRs as “low risk.” Regarding PRISMA, four of the eight SRs were rated over 50%. Based on the GRADE tool, two out of six SRs rated maternal depressive symptoms as “moderate;” one out of five SRs rated paternal depressive symptoms as “moderate;” one out of six SRs estimated family functioning as “moderate,” and the other evidence was rated as “very low” or “low.” Of the eight SRs, six (75%) reported that maternal depressive symptoms were significantly reduced, and two SRs (25%) were not reported.

**Conclusion:**

Family-centered interventions may improve maternal depressive symptoms and family function, but not paternal depressive symptoms. However, the quality of methodologies, evidence, reporting, and bias of risk in the included SRs of family-centered interventions for perinatal depression was not satisfactory. The above-mentioned demerits may negatively affect SRs and then cause inconsistent outcomes. Therefore, SRs with a low risk of bias, high-quality evidence, standard reporting, and strict methodology are necessary to provide evidence of the efficacy of family-centered interventions for perinatal depression.

## Introduction

Perinatal depression, an important mental health problem, adversely affects birth outcomes, which can result in poor maternal–infant and father–infant interactions and increase the risk for child maltreatment (1–3). Patients may suffer from depressive symptoms anytime within the first year after the birth of children or even during pregnancy. Research has shown that perinatal depression affects 15 to 35% of mothers (4). In addition, considering the present COVID-19 pandemic, related studies have revealed an increase in the prevalence rate of perinatal depression in females (5, 6).

The improvement in families regarding depressive symptoms and dyadic adjustment is relevant, and fathers' needs for psychological resources have increased. In particular, if they support a partner with perinatal depression, then their vulnerability to anxiety, depression, and stress will be heightened (7, 8). A meta-analysis has presented that the prevalence rate of paternal perinatal depression is 10.4%, and it increases from 24 to 50% in the case of concomitant depression in the partner (9). Therefore, interventions for perinatal depression that address the mother and father may improve family outcomes (10).

Research has suggested that the adverse factors of perinatal depression can affect other adult family members, and they can cause apparent psychological disturbances and burdens (11–14). Some scholars have published some reports on intergenerational families. In particular, the poor coparenting relationship between a parent and grandparent could negatively affect their psychological health (15–17). On the contrary, a better transition from children to parenthood could be experienced if couples obtained positive support from the grandparents (18). With regard to the interrelation between the perinatal mental health of a woman and her family, the results may be optimized by interventions of perinatal depression, including the woman and her partner or the major supporter in the family (19, 20). Family-centered intervention has shown great application potential in preventing perinatal depression or supporting its recovery (21).

Family-centered intervention can be considered as any psycho-therapeutic endeavor that focuses on changing the interaction among family members and promotes family function as a unit or subsystems or/and the function of the individual members of the family (22). In addition, family-centered interventions for depression help participants and their families disengage from destructive communication modes and reduce depressive symptoms (23). Based on the efficacy of family-centered interventions on perinatal depression, previous studies have shown that family-centered intervention is effective for the mental health of the family (24, 25).

As major public health strategies, the development of accessible family-centered interventions can reduce the effect of the adverse outcomes of perinatal mental health issues on children and parents (26, 27). However, based on searched systematic reviews (SRs) in this paper, a large number of studies have varied in intervention types, duration, and results. The qualities of these SRs and meta-analysis are not evaluated, whereas the evaluation cannot be ignored before treatment recommendations can be provided confidently.

Our research aimed to perform a comprehensive overview of the quality of the methodology, risk of bias, reporting, and evidence in these SRs in order to evaluate the available evidence regarding the effect of family-centered interventions on perinatal depression.

## Methods

### Design

This paper was designed for the overview of SRs (OoSRs). The ethical protocol was not needed to be prepared due to the nature of this study. The Cochrane Handbook for SRs of Interventions (the part of overview) gave some guidance for our paper, and Preferred Reporting Items for OoSR (PRIO-harms) statement reported our research (28, 29). The protocol for this paper was prospectively registered with PROSPERO (CRD42021290611).

### Inclusion and exclusion criteria

The PICOS (population, intervention, comparison, outcomes, and study) was used to perform a precise search strategy.

#### Population

Mothers who are pregnant or postpartum for up to a year and have at least one adult family member enrolled. The family is defined as a unit composed of family members. The family member was defined as the person biologically related to the mother or a close person she thought but without consanguinity (30).

#### Intervention

This review included studies that addressed couple relational dynamics, coparenting, or dynamics involving extended family members/next of kin, was performed in many forms such as cognitive-behavioral skills training, behavioral marital therapy, solution-focused therapy, and interpersonal therapy. Interventions related to the parents of hospitalized premature infants were excluded. Restrictions were not set on the implementation environment or time of the intervention.

#### Outcomes

Relevant outcome measures were predefined as follows: (a) Symptoms of maternal depression; (b) Symptoms of paternal depression; (c) Family functioning: coparenting relationship, satisfaction with the couple relationship, partner relationship quality, and so on.

The inclusion criterion was as follows: at least one data of the abovementioned outcomes of interest must be reported by SRs.

#### Studies

All non-Cochrane SRs and Cochrane SRs of non-randomized and randomized-controlled clinical trials (RCTs) were enrolled in this study. Experimental evidence was collated, and our prespecified criteria were satisfied. In our criteria, a specific research question was answered by a systematic review, and the bias was minimized using systematic and explicit methods. Then, reliable findings were drawn from the conclusions (31).

### Search means

Searched electronic bibliographic databases were presented as follows: Cochrane Library, Embase, PubMed, CINAHL, China National Knowledge Infrastructure, Web of Science, PsycINFO, VIP database, and Wan Fang database from the establishment of the database to December 31, 2022. Articles published in Chinese or English were enrolled. Table 1 shows the search strategy. In addition, gray literature and references such as Google Scholar, ProQuest Health, and Medical Collection were searched.

**Table 1:** Search terms.

S1	Pregnanc * OR prenatal OR postpartum OR antenatal OR postnatal OR Peripartum OR puerperal OR perinatal OR primiparous OR multiparous
S2	Famil * OR parent * OR paternal * OR caregiver * OR spous*
S3	Depress * OR mood disorder
S4	Psychother * OR psycho-therap* OR family therapy OR family intervention OR family systems OR family based OR marital therapy OR couple therapy OR co-parenting
S5	Meta analysis OR meta-analysis * OR meta analysis * OR systematic * OR review * OR overview * OR synthesis * OR integrative research review * OR research integration
S6	S1 AND S2 AND S3 AND S4 AND S5

### Data management and data collection

Data management was performed by Mendeley. The titles were independently screened, and potentially relevant studies were abstracted by two researchers (HP/YX) after removing duplication. Based on the exclusion and inclusion criteria, the full texts of eligible studies were read and downloaded. Afterward, a cross-check was conducted to avoid midentry. A third reviewer (SK) was involved in the discussion of discrepancies.

Two researchers (HP/YX) independently completed data extraction. The third reviewer (SK) participated in the discussion of discrepancy. The data was collected in advance by using a developed extraction form. In addition, the extracted data included the following contents:

- Study features (number of included studies, study types, interventions, quality of assessment tools, data analysis methods).
- General information (author, title, language, and country).
- Results.
- Conclusion summary.

### Methodological quality evaluation

AMSTAR 2 is a tool commonly applied in evaluating the methodology of SRs (32). Thus, AMSTAR 2 was adopted by two researchers (HP/YX) to independently assess the methodology of SRs. A total of 16 items were included in AMSTAR 2, including seven key items (items 2/4/7/9/11/13/15) that could significantly affect the effectiveness of an SR and its conclusion. Based on the AMSTAR 2 guideline criterion, “yes,” “partial yes,” and “no” served as evaluation modes. Furthermore, team discussion was performed to solve discrepancies.

### Bias risk evaluation

ROBIS, an instrument for evaluating the risk of bias in SRs (33), consists of three stages formed by signaling questions. The bias risk in each SR was independently appraised by two researchers (HP/YX) using ROBIS. Phase 1 was optional, and it primarily assessed relevance. In addition, four domains formed by 21 signaling questions was composed of phase 2, which was responsible for identifying issues during review. Three signaling questions were involved in phase 3, wherein the bias risk of SRs was determined. All signaling questions were answerable by “no,” “probably no,” “no information,” “probably yes,” and “yes.” The answer “yes” for signaling questions in phase 3 indicated SR with a “low risk” of bias. The answer “probably no” or “no” in phase 3 indicated a “high risk” assessment in SR. Moreover, SR with insufficient information would be rated as “unclear risk.” Team discussions were required to solve discrepancies.

### Reporting quality assessment

Preferred Reporting Items for Systematic Reviews and Meta-Analyses (PRISMA), consisting of a four-phase flow diagram and a 27-item checklist, is a reporting guidance for improving the transparency of SRs (34–36). PRISMA was used by two researchers (HLP/YJX) to independently assess the quality of reporting. In addition, 27 was considered as the highest score. Each item was appraised on the basis of whether or not it was reported. In particular, a full report could be given one point, an incomplete report for 0.5 points, and blank report for 0 point. Points fewer than 15 indicated relatively terrible defects in the information of report; 15–21 points indicated the lack of reports, and 21.5–27 points indicated a relatively complete report (37).

### Evaluation of evidence quality

Evidence quality of each SR result was commonly rated through the grading of recommendations, assessment, development, and evaluations (GRADE) (38). For the included SRs, GRADE was adopted by two researchers (HLP/YJX) to independently assess the quality of each outcome, including maternal depressive symptoms, paternal depressive symptoms, and family functioning. Based on GRADE, observational studies were of low quality, and evidence based on RCTs was of high quality at the beginning, whereas the evidence quality might decline by the five key factors of GRADE (inconsistency, publication bias, imprecision, indirectness, and bias risk). All evidence qualities of outcomes were graded as “very low,” “low,” “moderate,” and “high.”

### Presentation and synthesis of data

Tabulations were utilized to summarize the features of included SRs and the outcomes of ROBIS, AMSTAR 2, GRADE, and PRISMA. Given the heterogeneity and the absence of data consolidation in some SRs, the effectiveness of family-centered interventions on perinatal depression was narratively summarized.

## Results

### Search outcome

**Figure 1:**
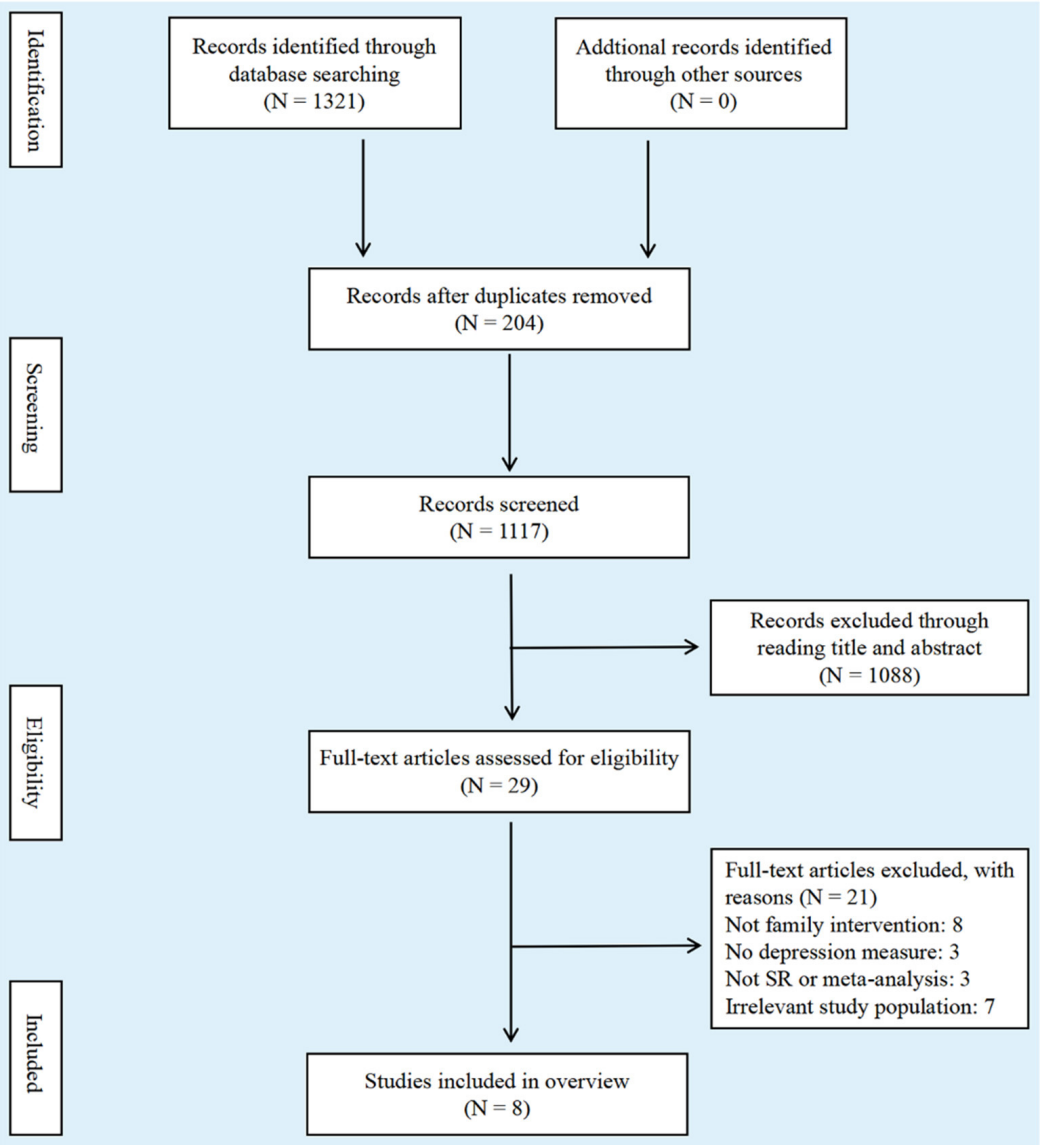
Flow diagram of literature search.

A total of 1,321 papers were retrieved, of which 1,117 remained after removing duplicates. The summary and title were browsed, and 29 potentially relevant reviews were determined. After reading the full text, 21 studies were removed and 8 reviews were retained. Figure 1 shows a flow diagram of the literature search.

### Features of the enrolled reviews and overlap between the included reviews

Table 2 shows the features of enrolled reviews after summary. The publication dates of all literature were from 2015 to 2021, including one article (45) from Australia, one (40) from Ireland, two (21, 42) from America, two (39, 44) from Japan, one (43) from Portugal, and one (41) from China. Four SRs (50%) (21, 41, 44, 45) included RCTs only, and the remaining four (50%) (39, 40, 42, 44) included RCTs and non-RCTs. Furthermore, all SRs were published in English. The included 8 reviews contained a total of 79 primary studies, of which 19 were included in more than one review. The overlap results showed that this overviews had low covered area (CA) (0.1598) and low corrected covered area (CCA) (0.0398) with 24.05% overlap, indicating a slight overlap across the included reviews in terms of their primary studies.

**Table 2:** Overview of key characteristics of included reviews.

**Author/year/country**	**Language**	**Type of studies**	**Number studies (participants)**	**Overlap studies (n=19)**	**Methodology quality assessment**	**Intervention**	**Data analysis means**	**Outcomes**	**Author conclusion**
Iwata et al. (39) Japan	English	RCT, no-RCT, before-and-after, time-series, and cohort studies	4 (652)	1	JBI SUMARI	Educations for expectantly primiparous couples and females		Depressive symptoms	Parenting education content that would be effective in preventing depressive symptoms in pregnant women.
Noonan et al. (40) Ireland	English	RCT, quasi-experimental and post-test /pre-test studies	9 (509)	5	NHLBI	Psychosocial interventions involved a family member/partner		Depressive symptoms; relationships with their partner or family functioning	The family inclusion is vital in giving emotional and practical help to the female, and can contribute to normarize the relation of families and communities.
Xiao and Loke (41) China	English	RCT	12 (3650)	3	The Cochrane Risk of Bias Evaluation tool	Intergenerational co-parenting/ co-parenting interventions (psycho-education, skills training, communication, group discussions )	Meta-analysis	Co-parenting relationship; depressive symptoms	Some good effects on depression symptoms are demonstrated by co-parenting interventions in co-parenting undermining, co-parenting support, couple communication, and interactions of parents, parent-child and mothers.
Cluxton-Keller et al. (21) America	English	RCT	7 (801)	5	The Cochrane Collaboration's Risk of Bias	Family therapeutic interventions ( behavioral marital therapy, CBT,IPT and solution-focused therapy)	Meta- analysis	Depressive symptoms; family functioning	Perinatal depressive symptoms are obviously decreased after family interventions, but intervention intensity (treatment, indicated or universal) affects the efficacy.
Lee et al. (42) America	English	RCT, quasi-randomized, quasi-experimental, non-experimental, and qualitative studies	21 (1366)	3	RoBANSs	Parent education programs		Partner relationship quality	Results concerning partner relationship quality, coparenting relationship, father's mental health and father involvement may be improved by early father-inclusive parent educations
Alves et al. (43) Portugal	English	RCT, quasi-experimental studies	24 (842)	10	NCCMT	no-biological interventions targeted both member of the couple or just the woman (CBT, education,psychosocial ,IPT)		Depressive symptoms	Interventions involved the partner have great benefits in treating or preventing female's postpartum depression. Nevertheless, the imformation on the specific behavior and applicable ways to utilize partners to imrove intervention effucacy were unclear.
Suto et al. (44) Japan	English	RCT	11 (5000)	5	Cochrane Handbook for Systematic Reviews of Interventions	Education programs for partners of pregnant women		Depressive symptoms; satisfaction with the postnatal couple relationship	Evidence-based prenatal education programs are understood systematically to support expectant couples to transit to parenthood.
Pamela et al. (45) Australia	English	RCT	13 (2087)	9	Cochrane Handbook for Systematic Reviews of Interventions	Partner-inclusive interventions (psycho-educational approaches, couple-focused interventions, home visits or telephone support)		Depressive symptoms	Except for improving paternal results, research indicated that partner inclusion also improved interventions' compliance in mother.

NHLBI, National Heart, Lung and Blood Institute; JBI SUMARI, JBI System for the Unified Management, Assessment and Review of Information; RoBANSs, The Risk of Bias Assessment tool for no-randomized Studies; NCCMT, National Collaborating Center for Methods and Tools; CBT, Cognitive-behavioral therapy; IPT, Interpersonal psychotherapy.

Five reviews (21, 41, 43–45) evaluated literature qualities through Cochrane risk of bias, and one review (39) utilized the JBI System for the Unified Management, Assessment, and Review of Information (JBI SUMARI). In addition, one review (40) used quality assessment tools for National Heart, Lung, and Blood Institute, and one review (42) adopted the risk of bias assessment tool for nonrandomized studies. Only two reviews (21, 41) used meta-analysis in data synthesis. Each SR involved 4–24 articles, and 509–5000 subjects participated in this study. Family-centered interventions were primarily explored in our paper, including psycho-education, skills training, and communication.

### Quality of included reviews

#### Methodological quality results

The outcomes of the methodological quality of SRs treated by AMSTAR 2 are displayed in Table 3. Five SRs (40–43, 45) were of very low quality, and three SRs (21, 39, 44) were of low quality. The items with the lowest compliance rates in AMSTAR 2 (*i.e*., lower rates of “√”) included I3 (Did the review authors explain their selection of the study designs for inclusion in the review? 12.5%), I2 (“a prior protocol provided,” 50%), I10 (Did the review authors report on the sources of funding for the studies included in the review? 0%), and I7 (Did the review authors provide a list of excluded studies and justify the exclusions? 25%). Given the absence of meta-analysis in six SRs (39–42, 44, 45), three critical items (I11, I12, and I15) showed low rates of “√.”

**Table 3:** Methodological quality of included reviews.

**Questions**	**Author (year)**							
	**Iwata et al**. **(****39****)**	**Noonan et al**. **(****40****)**	**Xiao and Loke** **(****41****)**	**Cluxton-Keller et al**. **(****21****)**	**Lee et al**. **(****42****)**	**Alves et al**. **(****43****)**	**Suto et al**. **(****44****)**	**Pamela et al**. **(****45****)**
Q1	√	√	√	√	√	√	√	×
Q2^a^	√	×	×	√	√	×	√	×
Q3	×	×	×	×	√	×	×	×
Q4^a^	!	!	!	!	!	!	!	!
Q5	√	√	√	√	×	√	√	×
Q6	√	√	√	×	×	√	×	√
Q7^a^	√	×	×	×	×	×	√	×
Q8	√	√	√	√	√	√	√	√
Q9^a^	√	√	√	√	√	√	√	√
Q10	×	×	×	×	×	×	×	×
Q11^a^	0	0	√	√	0	0	0	0
Q12	0	0	√	√	0	0	0	0
Q13^a^	√	√	√	√	√	√	√	×
Q14	√	√	√	√	√	√	√	√
Q15^a^	0	0	√	√	0	0	0	0
Q16	√	√	√	√	√	√	√	√
Quality rating	Low	Critically low	Critically low	Low	Critically low	Critically low	Low	Critically low

^a^Critical items. 0, no meta-analysis; Q, signaling questions; √, yes; ×, no; !, partial yes. Q1-Q16: See Appendix A. Methodological quality of reviews according to the criteria: (1) Critically low: more than one critical defect with or without non-critical demerits; (2) Low: one key shortcoming with or without non-critical flaws; (3) Moderate: over one non-critical demerit, multiple non-critical weaknesses may decrease confidence in the review, and the overall appraisal confidence might be moved down from moderate to low level; 4.High: no or one non-critical weakness.

### Results of the bias risk

Table 4 shows a low risk in all SRs in phase 1. For phase 2, the low risk of domain 4, domain 3, domain 2, and domain 1 was 37.5%, 25%, 37.5%, and 100%, respectively. For phase 3, the low risk was 50%. In addition, ROBIS's signaling questions (Q) with the highest concerns (i.e., higher rates of “no”) included Q7 (Were methods except for database searching applied to check relevant articles? no = 50%), Q6 (Did the search involve an available range of electronic sources/databases for unpublished and published literature? no = 37.5%), Q22 (Did explanations of findings solve all concerns scanned in domains 1 to 4? no = 37.5%), and Q21 (Were biases in primary studies minimal or addressed in the synthesis? no = 37.5%). On the basis of the final ROBIS phase, four (21, 39, 41, 42) out of eight SRs were considered as “low risk,” three (40, 43, 45) were considered as “high risk,” and one (44) was considered as “unclear risk.”

**Table 4:** The result of ROBIS.

**Signaling questions of ROBIS**	**Author (year)**							
	**Iwata et al**. **(****39****)**	**Noonan et al**. **(****40****)**	**Xiao and Loke** **(****41****)**	**Cluxton-Keller et al**. **(****21****)**	**Lee et al**. **(****42****)**	**Alves et al**. **(****43****)**	**Suto et al**. **(****44****)**	**Pamela et al**. **(****45****)**
1.1	√	√	√	√	√	√	√	√
2.1.1(Q1)	√	!	!	√	√	!	√	!
2.1.2(Q2)	√	√	√	√	√	√	√	!
2.1.3(Q3)	√	√	√	√	√	√	√	!
2.1.4(Q4)	√	!	!	√	!	√	√	!
2.1.5(Q5)	√	!	!	!	√	!	√	!
Concerns regarding specification of study eligibility criteria	Low	Low	Low	Low	Low	Low	Low	Low
2.2.1(Q6)	√	√	√	√	√	×	×	×
2.2.2(Q7)	√	×	!	×	×	√	×	√
2.2.3(Q8)	√	!	!	√	√	!	!	×
2.2.4(Q9)	!	×	√	!	√	√	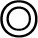	×
2.2.5(Q10)	√	√	√	√	0	√	√	×
Concerns regarding methods used to identify and/or select studies	Low	High	Low	Low	Unclear	High	High	High
2.3.1(Q11)	√	√	√	×	0	√	0	√
2.3.2(Q12)	√	√	√	√	√	√	√	√
2.3.3(Q13)	√	×	!	√	√	!	√	×
2.3.4(Q14)	√	√	√	√	√	×	√	√
2.3.5(Q15)	√	√	√	√	0	0	√	√
Concerns regarding methods	Low	High	Low	High	Unclear	Unclear	Unclear	High
2.4.1(Q16)	√	√	√	√	√	√	√	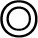
2.4.2(Q17)	√	0	0	√	√	!	√	0
2.4.3(Q18)	√	√	!	√	√	√	√	√
2.4.4(Q19)	√	√	√	√	√	√	√	√
2.4.5(Q20)	√	!	!	√	!	!	!	×
2.4.6(Q21)	√	!	!	√	×	×	√	×
Concerns regarding the synthesis and findings	Low	Unclear	Unclear	Low	High	High	Low	High
3.1(Q22)	√	×	√	√	√	×	0	×
3.2(Q23)	√	√	√	√	√	√	√	√
3.3(Q24)	√	√	√	√	√	√	√	√
Risk of bias in the review	Low	High	Low	Low	Low	High	Unclear	High

Q, signaling questions; ×, no; √, yes; !, probably yes; 0, no information; 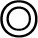
probably no Questions of ROBIS: See Appendix B.

### Outcomes of reporting quality

Table 5 shows the PRISMA results. The mean score was 19.7 (range 12.5–24.5) after reporting quality evaluation. In particular, one review (45) received ≤15 points; three (21, 42, 43) received 15–21 points, and four (39–41, 44) received >21 points. Fewer points (*i.e*., lower rates of “√”) were observed in certain items. These items included I22 (risk of bias across literature, 6.25%), I15 (risk of bias across research, 6.25%), I23 (additional analyses, 18.75%), and I16 (other analyses, 0%). Attention should be paid to these items because they might involve major reporting limitations.

**Table 5:** Reporting quality assessment of systematic reviews by PRISMA.

**Section**	**Items**	**Author (year)**							
		**Iwata et al**. **(****39****)**	**Noonan et al**. **(****40****)**	**Xiao and Loke** **(****41****)**	**Cluxton-Keller et al**. **(****21****)**	**Lee et al**. **(****42****)**	**Alves et al**. **(****43****)**	**Suto et al**. **(****44****)**	**Pamela et al**. **(****45****)**
Title	I1 Title	√	!	√	√	√	√	√	!
Abstract	I2 Structured summary	√	!	!	√	√	!	√	!
Introduction	I3 Rationale	√	√	√	√	√	√	√	√
	I4 Objectives	√	√	√	√	√	√	√	√
Methods	I5 Protocol and registration	√	×	×	√	√	√	√	×
	I6 Eligibility criteria	√	√	√	√	√	√	√	!
	I7 Information sources	√	!	√	√	!	√	√	√
	I8 Search	√	√	×	√	×	×	√	×
	I9 Study selection	√	√	√	√	×	√	√	!
	I10 Data collection process	!	!	√	!	×	!	×	!
	I11 Data items	!	!	√	!	√	×	×	!
	I12 Risk of bias in individual studies	√	√	√	√	√	×	√	!
	I13 Summary measures	√	√	×	√	√	×	√	×
	I14 Synthesis methods	√	√	√	√	√	!	√	×
	I15 Risk of bias across studies	×	×	×	√	!	×	×	×
	I16 Additional analyses	×	×	×	√	×	×	×	×
Results	I17 Study selection	√	√	√	√	√	√	√	√
	I18 Study characteristics	√	√	√	√	√	√	√	√
	I19 Risk of bias within studies	√	√	√	√	√	√	√	×
	I20 Results of individual studies	√	!	√	√	√	!	√	!
	121 Synthesis of results	√	×	√	√	√	!	√	×
	I22 Risk of bias across studies	×	×	√	×	√	×	!	×
	I23 Additional analyses	×	×	√	!	×	×	×	×
DISCUSSION	I24 Summary of evidence	√	√	√	√	√	√	√	√
	I25 Limitations	√	√	√	√	√	√	√	!
	I26 Conclusions	√	√	√	√	√	√	√	√
Funding	I27 Funding	√	√	√	√	√	√	√	√
Total score		23	18	21.5	24.5	21	15.5	21.5	12.5

√, yes; ×, no; !, partial yes.

### Results with GRADE

Evidence of maternal depressive symptoms in two SRs (21, 41) (2/6, 33.34%) was rated as “moderate.” Paternal depressive symptoms in one SR (41) (1/5, 20%) and family functioning in one SR (21) (1/5, 20%) were also rated as “moderate.” These results are presented in Table 5. In addition, family-centered interventions must include a partner/family member, but the group allocation could not be ignored by participants. This limitation may be due to the loss of blinding, which results in data bias. Therefore, all RCT articles were graded as “serious (−1)” in the bias risk category. Moreover, inconsistent format and contents of interventions for the same outcome might cause few high-quality evidence in our article. Therefore, a rigorous and comprehensive SR is necessary to prove the availability of family-centered interventions for perinatal depression.

### Effectiveness of family-centered interventions

#### Maternal depressive symptoms

Of the eight SRs (21, 39–41, 43, 45), six (75%) reported that maternal depressive symptoms were significantly reduced, and two SRs (25%) (42, 44) were not reported. One SR (12.5%) (21) reported that the depressive symptoms in mothers who received an indicated prevention intervention and treatment were evidently reduced compared with those who received general prevention interventions, and depressive symptoms of family members of mothers who participated in at least 80% of the intervention treatment decreased significantly.

#### Paternal depressive symptoms

Meta-analysis from Xiao and Loke (14) indicated that family-centered interventions had no effect on paternal symptoms (95% CI −0.22 to 0.11, I^2^ = 0%, *P* = 0.52, z = 0.69, *P* = 0.49), based on the SRs of Suto et al. (44). However, the results obtained by Pamela et al. (45), Noonan et al. (40), and Lee et al. (42) indicated the benefit of family-centered interventions on paternal depression symptoms.

#### Family functioning

The information presented in our report was insufficient to identify the efficacy of family-centered intervention on family functioning, as shown by the results of two SRs (40, 44). However, family involvement is vital for providing women with emotional and practical support, which can improve health outcomes. This finding is supported by other four SRs (21, 41, 42, 45) (Table 6).

**Table 6:** Quality of evidence.

**Outcomes**	**Author**							
	**Iwata et al**. **(****39****)**	**Noonan et al**. **(****40****)**	**Xiao and Loke** **(****41****)**	**Cluxton-Keller et al**. **(****21****)**	**Lee et al**. **(****42****)**	**Alves et al**. **(****43****)**	**Suto et al**. **(****44****)**	**Pamela et al**. **(****45****)**
Maternal depressive symptoms	Very low	Very low	Moderate	Moderate	–	Low	–	Very low
Paternal depressive symptoms	–	Very low	Moderate		Very low	–	Low	Very low
Family functioning	–	Very low	Low	Moderate	Very low	–	Low	Very low

Therefore, family-centered interventions can reduce maternal depressive symptoms, but they have uncertain effects on paternal depressive symptoms. The results revealed a trend for family functioning improvement.

## Discussion

### Main findings

In this study, we included SR results and evaluated the quality of methodology, evidence, reporting, and risk of bias. In particular, AMSTAR 2 showed five SR methodologies with critically low quality and three with low quality. ROBIS revealed the bias risk of three SRs rated as “high,” one as “unclear,” and four as “low.”

In addition, the PRISMA checklist exhibited relatively great reporting quality in most of the SRs (50%). However, the quality of these SR in terms of reporting, evidence, methodology, and risk of bias for family-centered interventions of perinatal depression remained unsatisfactory. These demerits weakened evidence reliability for family-centered interventions of perinatal depression. Therefore, we should act prudently when suggesting family-centered interventions as a treatment or prevention for promoting family function and reducing perinatal depression.

In this report, we focused on the qualities of reporting, risk of bias, and methodology in SRs about family-centered interventions for perinatal depression. Some potential repetitions in partial items of PRISMA, ROBIS, and AMSTAR 2 were discovered. Based on the results, the following common shortcomings in the included SRs were stressed: (1) there was slight overlap across the included reviews in terms of their primary studies, (2) the absence of some additional methods and comprehensive strategies for literature search, (3) the lack of additional forward-looking description analyses in methods, and (4) a suitable method to check the robustness of the findings and address heterogeneity.

### Implication for future study

#### For clinicians

First, based on the results of our study, we found that family-centered intervention could improve maternal depressive symptoms and family function, rather than paternal depressive symptoms. This finding is consistent with previous outcomes, that is, research in interventions for paternal depression is lacking (7). In most studies, paternal wellbeing was addressed indirectly through concentrating on the infant, mother, or conjugal relation rather than exclusively targeting paternal mental health (46). For ordinary adults who are becoming parents, fathers' support, education, and involvement might decrease depression scores. A great effect on the family can be obtained by studying effective interventions in treating paternal depression.

Second, given the diverse interventions and bias, the evidence quality was primarily very low or low. The future report should stress assessors' blinding and allocation concealment. In addition, several results were reported by mothers or family members spontaneously, and they could not blind themselves to the test. The reliability of the included studies may be enhanced when the intervention outcomes are evaluated with the combination of diagnosis methods and self-report in measurements (47, 48).

#### For the author of further SR

If producers emphasize on standard reporting, bias risk, and methodology quality of SRs, then these defects can be averted. Thus, some advice is provided for SR producers.

First, SR should be registered on registration platforms such as the international database PROSPERO (https://www.crd.york.ac.uk/prospero/). Based on these reports, advance registration may contribute to SR performance, design, and reporting quality (49–51). Second, additional searching methods are important for producers of SRs to obtain more eligible articles. Third, the methods such as contacting experts, citation searches, hand-searching, and reference checking are recommended. The excluded studies must be listed, or the influence of their exclusion might be unclear because they are invisible. Finally, additional analyses of statistical methods should be described prospectively, including analysis of meta-regression, subgroup, and sensitivity.

## Merits and limitations

Our paper showed a few merits. First, this paper is the first overview that completely assessed the SRs of family-centered interventions for perinatal depression through GRADE, PRISMA, ROBIS, and AMSTAR 2. There were no new related systematic reviews have been published since our search was conducted. In addition, we could comprehensively understand the effectiveness of family-centered interventions on perinatal depression in clinical practice. Moreover, the current high-quality evidence for SRs was combined to provide more convincing evidence in clinical practice.

Moreover, some limitations were not overlooked. On the one hand, only studies in Chinese and English were searched; thus, the information was not complete. On the other hand, part of the included SRs was overlapping. Thus, the effectiveness of family-centered interventions for perinatal depression was only described narratively, and raw RCT data included in available SRs were not synthesized.

## Conclusion

SRs on family-centered interventions for perinatal depression are increasing. Nevertheless, by appraising the SRs on family-centered interventions for perinatal depression, these SRs have suboptimal quality in evidence, risk of bias, methodology, and reporting. Therefore, SRs with a more logical methodology, high-quality evidence, prescriptive reporting, and less risk of bias are necessary to furnish family-centered interventions for perinatal depression with compelling evidence.

## Data availability statement

The original contributions presented in the study are included in the article/Supplementary material, further inquiries can be directed to the corresponding authors.

## Author contributions

Research idea and study design: LH and KS. Data acquisition: JY and XD. Data analysis/interpretation: KS and XD. Manuscript drafting and revision: LH and AC. All authors read and approved the final manuscript.
