# Supplementary material for: Effect of family-centered interventions for perinatal depression: an overview of systematic reviews
Source: Front Psychiatry. 2023 Jun 1;14:1094360. doi: 10.3389/fpsyt.2023.1094360 (PMC10267375; doi:10.3389/fpsyt.2023.1094360)
Supplement: Supplementary file 2 [file Data_Sheet_2.doc]

Supplementary file 2 Phase 1:assessing relevance

1.1 Does the question addressed by the review match the target question？

Phase2:identifying concerns about bias in the review process

2.1 Concerns regarding specification of study eligibility criteria

2.1.1(Q1) Did the review adhere to pre-defined objectives and eligibility criteria?

2.1.2(Q2) Were the eligibility criteria appropriate for the review question?

2.1.3(Q3) Were eligibility criteria unambiguous?

2.1.4(Q4) Were any restrictions in eligibility criteria based on study characteristics appropriate (eg, date, sample size, study quality, outcomes measured)？

2.1.5(Q5) Were any restrictions in eligibility criteria based on sources of information, appropriate (eg publication status or format, language, availability of data)?

2.2 Concerns regarding methods used to identify and/or select studies

2.2.1(Q6) Did the search include an appropriate range of databases/electronic sources for published and unpublished reports?

2.2.2(Q7) Were methods additional to database searching used to identify relevant reports?

2.2.3(Q8)Were the terms and structure of the search strategy likely to retrieve as many eligible studies as possible?

2.2.4(Q9) Were restrictions based on date, publication format, or language appropriate?

2.2.5(Q10) Were efforts made to minimise error in selection of studies?

2.3 Concerns regarding methods

2.3.1(Q11) Were efforts made to minimise error in data collection?

2.3.2(Q12) Were sufficient study characteristics available for both review authors and readers to be able to interpret the results?

2.3.3(Q13) Were all relevant study results collected for use in the synthesis?

2.3.4(Q14) Was risk of bias (or methodological quality) formally assessed using appropriate criteria?

2.3.5(Q15) Were efforts made to minimise error in risk of bias assessment?

2.4 Concerns regarding the synthesis and findings

2.4.1(Q16) Did the synthesis include all studies that it should?

2.4.2(Q17) Were all pre-defined analyses reported or departures explained?

2.4.3(Q18) Was the synthesis appropriate given the nature and similarity in the research questions, study designs and outcomes across included studies?

2.4.4(Q19) Was between-study variation (heterogeneity) minimal or addressed in the synthesis?

2.4.5(Q20) Were the findings robust, eg, as demonstrated through funnel plot or sensitivity analyses?

2.4.6(Q21) Were biases in primary studies minimal or addressed in the synthesis?

Phase 3: Judging risk of bias

3.1(Q22) Did the interpretation of findings address all of the concerns identified in Domains 1 to 4?

3.2(Q23) Was the relevance of identified studies to the review’s research question appropriately considered?

3.3(Q24) Did the reviewers avoid emphasizing results on the basis of their statistical significance?
