# Supplementary material for: Effect of family-centered interventions for perinatal depression: an overview of systematic reviews
Source: Front Psychiatry. 2023 Jun 1;14:1094360. doi: 10.3389/fpsyt.2023.1094360 (PMC10267375; doi:10.3389/fpsyt.2023.1094360)
Supplement: Supplementary file 3 [file Data_Sheet_3.docx]

|  | Hiroko Iwata (2021) | Maria Noonan (2021) | | Xiao Xiao (2021) | | Fallon Cluxton-Keller (2018) | | Joyce Y. Lee (2018) | | Stephanie Alves (2017) | | Maiko Suto (2016) | | Pamela D. PILKINGTON (2015) | |  |
| --- | --- | --- | --- | --- | --- | --- | --- | --- | --- | --- | --- | --- | --- | --- | --- | --- |
|  |  |  |  |  |  |  |  |  |  |  |  |  |  |  |  |  |
|  |  |  |  |  |  |  |  |  |  |  |  |  |  |  |  |  |
|  |  |  |  |  |  |  |  |  |  |  |  |  |  |  |  |  |
|  | Review 1 | | Review 2 | | Review 3 | | Review 4 | | Review 5 | | Review 6 | | Review 7 | | Review 8 |  |
| Abbass-Dick (2014) |  | |  | | x | |  | |  | |  | |  | |  |  |
| Alipour (2020) |  | | x | |  | |  | |  | |  | |  | |  |  |
| Barth (1988) |  | |  | |  | |  | | x | |  | |  | |  |  |
| Beal (1989) |  | |  | |  | |  | | x | |  | |  | |  |  |
| Bergström (2009) |  | |  | |  | |  | |  | |  | | x | |  |  |
| Bernard (2011) |  | |  | |  | |  | |  | | x | |  | |  |  |
| Brandon (2012) |  | | x | |  | |  | |  | | x | |  | |  |  |
| Brugha (2000) |  | |  | |  | |  | |  | | x | |  | |  |  |
| Bryan (2000) |  | |  | |  | |  | | x | |  | |  | |  |  |
| Buist (1999) |  | |  | |  | |  | |  | | x | |  | | x |  |
| Chen (2011) |  | |  | |  | |  | |  | | x | |  | |  |  |
| Cluxton-Keller (2018) |  | | x | |  | |  | |  | |  | |  | |  |  |
| Collado (2014) |  | | x | |  | | x | |  | |  | |  | |  |  |
| Daley-McCoy (2015) |  | |  | | x | | x | |  | |  | | x | |  |  |
| Danaher (2013) |  | | x | |  | |  | |  | | x | |  | |  |  |
| Diemer (1997) |  | |  | |  | |  | | x | |  | |  | |  |  |
| Doherty (2006) |  | |  | | x | |  | |  | |  | |  | |  |  |
| Doss (2014) |  | |  | | x | |  | |  | |  | |  | |  |  |
| Elliott (2000) |  | |  | |  | |  | |  | | x | |  | | x |  |
| Fagan (2008) |  | |  | |  | |  | | x | |  | |  | |  |  |
| Fawcett (1985) |  | |  | |  | |  | | x | |  | |  | |  |  |
| Fawcett (1987) |  | |  | |  | |  | | x | |  | |  | |  |  |
| Feinberg (2008) |  | |  | | x | | x | | x | |  | |  | | x |  |
| Feinberg (2009) |  | |  | |  | |  | | x | |  | |  | |  |  |
| Feinberg (2016) |  | |  | | x | |  | |  | |  | |  | |  |  |
| Feinberg (2020) |  | |  | | x | |  | |  | |  | |  | |  |  |
| Field (2008) |  | |  | |  | |  | | x | |  | |  | |  |  |
| Fisher (2010) |  | |  | |  | |  | |  | | x | |  | | x |  |
| Fisher (2016) |  | |  | |  | | x | |  | |  | |  | |  |  |
| Gambrel (2014) |  | |  | |  | | x | |  | |  | |  | |  |  |
| Gambrel (2015) |  | |  | |  | |  | | x | |  | | x | | x |  |
| Gao (2012) | x | |  | |  | |  | |  | |  | |  | |  |  |
| Gao (2010) |  | |  | |  | |  | |  | |  | |  | | x |  |
| Gjerdingen (2002) |  | |  | |  | |  | |  | |  | | x | |  |  |
| Halford (2010) |  | |  | | x | |  | |  | |  | |  | |  |  |
| Hart (1997) |  | |  | |  | |  | | x | |  | |  | |  |  |
| Hawkins (2006) |  | |  | |  | |  | |  | |  | | x | |  |  |
| Helnicke (1999) |  | |  | |  | |  | |  | |  | |  | | x |  |
| Hou (2014) |  | |  | |  | |  | |  | | x | |  | |  |  |
| Koushede (2017) |  | |  | | x | |  | |  | |  | |  | |  |  |
| Kozinszky (2012) |  | |  | |  | |  | |  | | x | |  | |  |  |
| Kuo (2009) | x | |  | |  | |  | |  | |  | |  | |  |  |
| Lane (2002) |  | |  | |  | |  | |  | | x | |  | |  |  |
| Lara (2010) |  | |  | |  | |  | |  | |  | |  | | x |  |
| Leung (2012) | x | |  | | x | |  | |  | |  | |  | |  |  |
| Li (2009) |  | |  | |  | |  | |  | |  | | x | |  |  |
| Mackert (2015) |  | |  | |  | |  | | x | |  | |  | |  |  |
| Mackert (2017) |  | |  | |  | |  | | x | |  | |  | |  |  |
| Mao (2012) |  | |  | |  | |  | |  | | x | |  | |  |  |
| Matthey (2004) |  | |  | |  | |  | |  | | x | | x | | x |  |
| Matthey (2008) |  | |  | |  | |  | |  | |  | |  | | x |  |
| Meager (1996) |  | |  | |  | |  | |  | | x | |  | |  |  |
| Melnyk (2006) |  | |  | |  | |  | |  | | x | |  | |  |  |
| Midmer (1995) |  | |  | |  | |  | |  | |  | | x | | x |  |
| Milgrom (2005) |  | |  | |  | |  | |  | | x | |  | |  |  |
| Milgrom (2011) |  | |  | |  | |  | |  | | x | |  | | x |  |
| Milgrom (2015) |  | |  | |  | |  | |  | | x | |  | |  |  |
| Milgrom (2016) |  | | x | |  | |  | |  | |  | |  | |  |  |
| Misri (2000) |  | |  | |  | | x | |  | | x | |  | |  |  |
| Morgan (1997) |  | |  | |  | |  | |  | | x | |  | |  |  |
| Mulcahy (2009) |  | | x | |  | | x | |  | |  | |  | |  |  |
| Mulcahy (2010) |  | |  | |  | |  | |  | | x | |  | |  |  |
| Ngai (2009) | x | |  | |  | |  | |  | |  | |  | |  |  |
| Petch (2012) |  | |  | | x | |  | |  | |  | |  | |  |  |
| Pfannenstiel (1991) |  | |  | |  | |  | | x | |  | |  | |  |  |
| Pfannenstiel (1995) |  | |  | |  | |  | | x | |  | | x | |  |  |
| Puckering (2010) |  | |  | |  | |  | |  | | x | |  | |  |  |
| Reay (2006) |  | |  | |  | |  | |  | | x | |  | |  |  |
| Salman-Engin (2017) |  | |  | |  | |  | | x | |  | |  | |  |  |
| Shapiro (2005) |  | |  | |  | |  | |  | |  | |  | | x |  |
| Shapiro (2011) |  | |  | | x | |  | |  | |  | |  | |  |  |
| Smith (1978) |  | |  | |  | |  | | x | |  | |  | |  |  |
| Smith (2016) |  | |  | |  | |  | | x | |  | |  | |  |  |
| Thomas (2014) |  | | x | |  | |  | |  | | x | |  | |  |  |
| Thome (2013) |  | | x | |  | |  | |  | |  | |  | |  |  |
| Tohotoa (2012) |  | |  | |  | |  | |  | |  | | x | |  |  |
| Van de Carr (1986) |  | |  | |  | |  | | x | |  | |  | |  |  |
| Westney (1988) |  | |  | |  | |  | | x | |  | |  | |  |  |
| Wöckel(2007) |  | |  | |  | |  | |  | |  | | x | |  |  |

**%Overlap=19/79=24.05**

**CA = (4+9+12+7+21+24+11+13) /(8*79)=0.1598**

**CCA = (101-79)/(632-79)=0.0398**
